# Effect of repeated bolus and continuous glucose infusion on a panel of circulating biomarkers in healthy volunteers

**DOI:** 10.1371/journal.pone.0279308

**Published:** 2022-12-27

**Authors:** Roland Feldbauer, Matthias Wolfgang Heinzl, Carmen Klammer, Michael Resl, Johannes Pohlhammer, Klemens Rosenberger, Verena Almesberger, Florian Obendorf, Lukas Schinagl, Thomas Wagner, Margot Egger, Benjamin Dieplinger, Martin Clodi

**Affiliations:** 1 Department of Internal Medicine, St. John of God Hospital Linz, Linz, Austria; 2 ICMR–Institute for Cardiovascular and Metabolic Research, Johannes Kepler Universität Linz (JKU Linz), Linz, Austria; 3 Department of Internal Medicine, Klinikum Rohrbach, Rohrbach, Austria; 4 Department of Laboratory Medicine, Ordensklinikum Linz, Linz, Austria; RCSI and MUB: Royal College of Surgeons in Ireland and Medical University of Bahrain, BAHRAIN

## Abstract

**Hypothesis:**

Glycaemic variability (GV) refers to fluctuations in the blood glucose level and may contribute to complications in patients suffering from Diabetes. Several studies show negative effects of GV on the cardiovascular system, however there is still a lack of conclusive evidence. Using an explorative cardiovascular panel, it is possible to simultaneously measure the effects on proteins relevant for cardiovascular processes. The aim of this study was to investigate the effects of rapid glucose excursions on cardiovascular and metabolic parameters in healthy individuals.

**Methods:**

An explorative single-blinded cross-over study was performed in ten healthy men. Subjects received 3 times 20 grams of glucose i.v. over 5 minutes or 60 grams of glucose continuously over 3 hours. Blood was taken for repeated measurements of the cardiovascular panel over the following 6 hours and again after 24 and 48 hours.

**Results:**

We observed a significant elevation of 7 cardiovascular biomarkers (BMP6, SLAMF7, LOX-1, ADAMTS13, IL-1RA, IL-4RA, PTX3) at t = 360min after rapid glucose infusion compared to a continuous glucose infusion.

**Conclusions:**

Intraday GV seems to have acute effects on cardiovascular proteins in healthy test persons. Rapid glucose administration compared to continuous administration showed significant changes in BMP6, SLAMF7, ADAMTS13, IL1RA, PTX3, IL-4RA and LOX-1.

**Clinical trial registration:**

NCT04488848.

## Introduction

At the end of the 20th century, it became known that people with diabetes have a 2 to 3 times higher risk of dying of cardiovascular death than non-diabetics. This has been displayed in epidemiological studies as well as in the United Kingdom Prospective Diabetes Study (UKPDS) [1, 2].

Today there are approximately 400 million people living with diabetes and about 1.5 million deaths are related to this disease every year [3].

In addition to the indisputable proofs that chronic hyperglycaemia plays a part in the pathogenesis of cardiovascular diseases and glucose lowering therapy may have a benefit, glycaemic variability (GV) has recently been regarded as another risk factor for cardiovascular and microvascular complications [4–7].

Furthermore, it was shown that GV cannot be adequately represented by HbA1c, the gold standard for assessment of glucose control [8].

The term glycaemic or glucose variability refers to fluctuations in the blood sugar level. Its cause is assumed to be reduced or a lack of self-regulation, or incorrect drug self-control. Intermittent blood sugar excursions with pronounced fluctuations between high and low values instead of constant, even increased blood sugar exposure, have turned out to be more harmful, according to several studies [9–14].

GV may therefore be a factor in the development of diabetic complications. Although many studies have shown negative effects of GV on the cardiovascular system, there is still a lack of conclusive evidence [15–22].

To our knowledge, there have to date been no studies with acutely altered glucose concentrations in healthy volunteers investigating the effects on a wide range of cardiovascular and metabolic biomarkers. New analytical techniques using explorative panels allow the identification of a broader range of new biomarkers and relevant protein signatures that may reflect important biological processes.

The cardiovascular panel used in this study provides simultaneous analysis of 92 protein biomarkers. Selection of protein biomarker assays is designed to focus on proteins relevant for cardiovascular processes. The assays in this panel include biomarkers involved in different biological processes which play a role in cardiovascular disease, such as inflammation, cellular metabolic processes, cell adhesion, immune response and complement activation.

The aim of this study is to determine the effect of rapid glucose excursion as a surrogate for high glucose variability compared to continuously elevated glucose levels on cardiovascular and metabolic parameters in healthy volunteers.

## Methods

### Study participants and design

**Fig 1:**
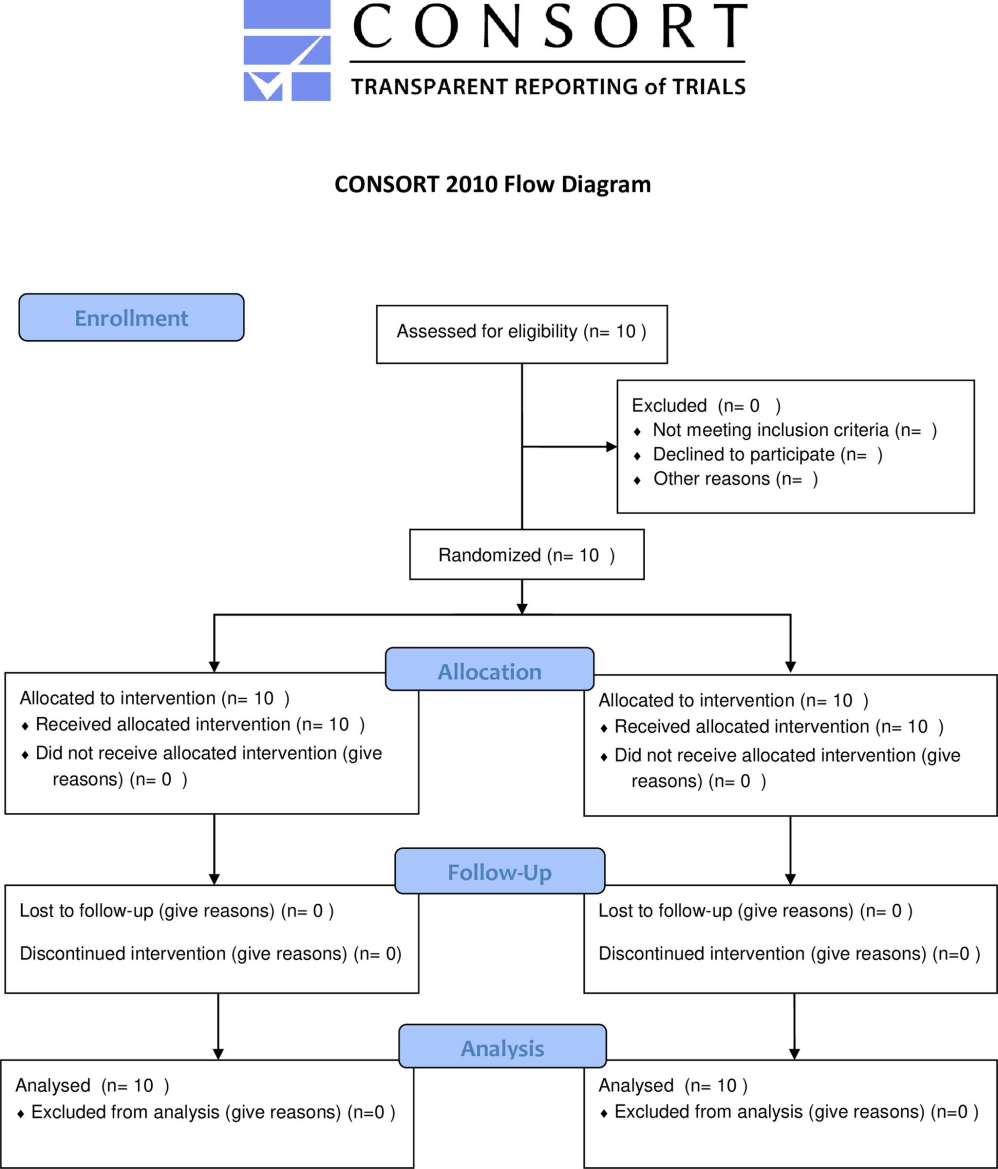
CONSORT flow diagram. The CONSORT Flow Diagram of the progress through the phases is depicted.

The study has been approved by the local joint research ethics committee of the St. John of God Hospital Linz. Informed consent was obtained in writing and orally from each subject before enrolment in the study. Ten healthy male volunteers were recruited via a notice at the local university (JKU Linz). After meeting the inclusion criteria (men aged 18 to 40 years, no disease history, no tobacco consumption, no diabetes history with fasting glucose and HbA1c within normal range) they were screened by medical history, physical examination, and electrocardiogram. Volunteers who suffered from any infectious disease or volunteers on any medication were excluded from the study (Fig 1). Baseline characteristics of the ten male volunteers can be found in Table 1.

**Table 1:** Baseline characteristics of volunteers.

Patient Code	Sex	Year of Birth	Age at Participation	Pulse ECG (bpm)	RR (mmHg)	Weight (kg)	BMI (kg/m²)	Waist circumference (cm)	Body Size (m)
B	Male	1988	30	67	115/73	73	23,84	87,5	1,75
A	Male	1997	22	54	133/75	100	30,86	110	1,8
K	Male	1994	24	70	138/87	75	23,41	82,5	1,79
G	Male	1992	26	55	115/71	79	24,93	88	1,78
C	Male	1998	21	63	125/79	78,4	23,93	81	1,81
i	Male	1996	23	53	107/76	78,5	25,93	92	1,74
E	Male	1993	26	88	121/73	94	27,76	87	1,84
M	Male	1993	26	54	124/74	86	27,14	90	1,78
D	Male	1991	28	57	107/59	92,2	25,54	90	1,9
H	Male	1993	27	57	116/68	77	22,50	90	1,85

In a cross-over design each subject was monitored on two different study days, 7–21 days apart. In random order, all subjects received 3 times 20 grams of glucose intravenously over 5 minutes at intervals of one hour on one day, as opposed to 60 grams of glucose continuously over 3 hours on the other study day. In between these study days subjects consumed a weight-maintaining diet providing at least 200 grams of carbohydrate each day.

As our study was single-blinded the medical staff involved in this study was informed about the infusion protocol and timepoints of venous blood sample collection. Participants of this study were blinded and received no information about the infusion protocol on each study day.

Study days began at 08.00 a.m. after an overnight fast and restraining from smoking or caffeine-containing beverages for 24 hours before the respective study day. Subjects then rested quietly in a supine position for the remainder of the study. Catheters (Safety iv Catheter with injection port; Braun, Melsungen, Germany) were inserted into both arms (one for sampling, the other for infusion).

Subjects received 3 times 20 grams of glucose dissolved in 100 ml of Aqua intravenously over 5 minutes in intervals of one hour (at time point t0, t60, t120), or 60 grams of glucose solved in 300 ml of Aqua continuously over 3 hours (starting at timepoint t0).

Blood was taken repeatedly for measurements of the Cardiovascular II panel (OLINK) (starting before the beginning of infusions at time point t0) over the following 6 hours and again after 24 hours and 48 hours.

Subjects were monitored consistently by a study assistant during the two study days including non-invasive blood pressure, heart rate and temperature.

### Laboratory measurements and statistical analysis

Venous blood samples were taken using VACUETTE polyethylene terephthalate glycol blood collection tubes (Greiner Bio-One). For measuring glucose levels the venous blood samples were analysed at the Department of Laboratory of the St. John of God Hospital Linz immediately, all other blood samples were centrifuged and stored.

EDTA-plasma samples (frozen at -80°C) were sent to OLINK Proteomics in Davos (Switzerland). By using the proximity extension assay (PEA), 92 biomarkers relevant for cardiovascular diseases were analysed. PEA works via matched pairs of antibodies carrying unique DNA tags that bind to the proteins in the probe. After binding to the protein DNA hybridization occurs. Using PCR amplification 96 biomarkers can be read out simultaneously [23, 24].

The concentrations of biomarkers are scaled in NPX (normalised protein expression) units. The NPX is Olink´s arbitrary unit on a log2-scale [25].

For statistical testing we used repeated-measures analysis of variance (RM-ANOVA). Whenever sphericity could not be assumed in RM-ANOVA (according to Mauchly´s test of sphericity) we used the Greenhouse-Geisser correction. When testing on singular timepoints we tested for normal distribution using Kolmogorov-Smirnov test. If data were normally distributed, we used paired t-test, otherwise we used Wilcoxon test.

For measuring glucose variability we used the coefficient of variation in our study. The coefficient of variation for each individual was calculated by dividing standard deviation through mean blood glucose which was then multiplied by 100 for receiving a percentage. We calculated the coefficient of variation for every test person on both study days.

Since this is an explorative study, no calculation of sample size was performed.

All calculations were performed using Statistical Package for the Social Sciences computer software (SPSS) in Version 26.

## Results

**Fig 2:**
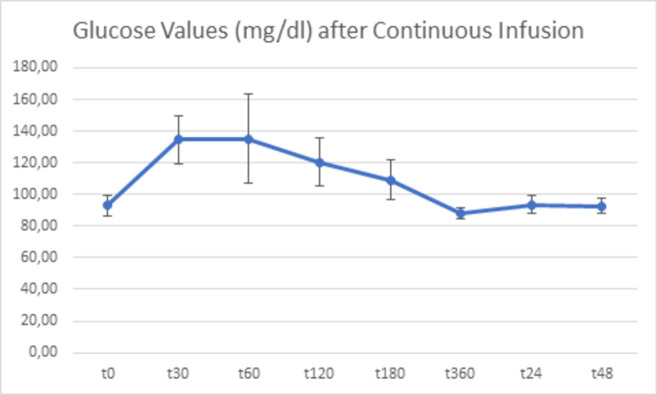
Blood glucose values (mg/dl) from t = 0 to t = 48h after continuous glucose infusion. Mean levels of blood glucose and standard deviation are depicted.

**Fig 3:**
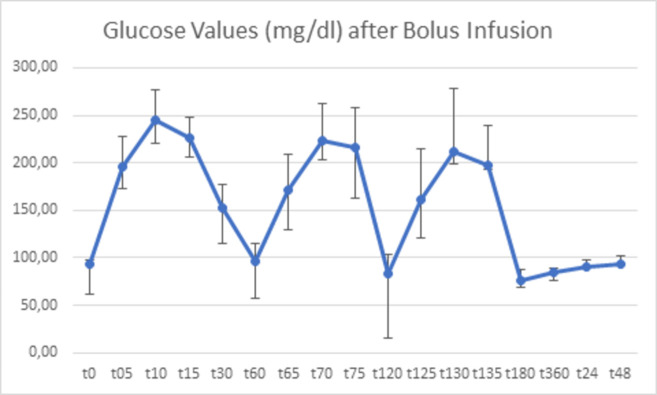
Blood glucose values (mg/dl) from t = 0 to t = 48h after bolus glucose infusion. Mean levels of blood glucose and standard deviation are depicted. Of note, additionally timepoints (t = 5min, t = 10min, t = 15min, t = 65min, t = 70min, t = 75min, t = 125min, t = 130min, t = 135min) are depicted.

**Fig 4:**
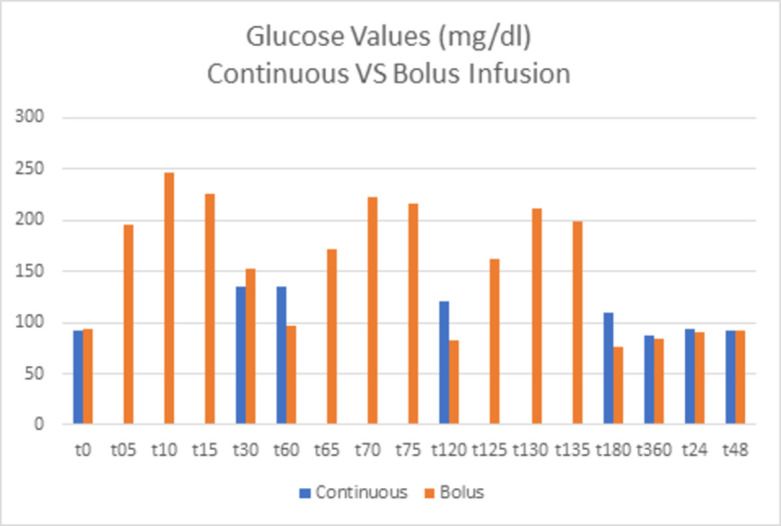
Blood glucose values (mg/dl) from t = 0 to t = 48h after continuous or bolus glucose infusion. Mean levels of blood glucose after continuous or bolus glucose infusion are depicted for each timepoint. Of note, additionally timepoints (t = 5min, t = 10min, t = 15min, t = 65min, t = 70min, t = 75min, t = 125min, t = 130min, t = 135min) for bolus infusion are depicted.

Blood glucose (in mg/dl) was measured on both study days (continuous vs bolus) (Tables 2 and 3). For better representation of glucose variability, additional glucose measurements were taken on days of bolus application at time points t = 5min, t = 10min, t = 15min as well as t = 65min, t = 70min, t = 75min and t = 125min, t = 130min, t = 135min (Figs 2–4).

**Table 2:** Descriptive statistics of blood glucose level after continuous infusion.

	N	MIn	Max	Mean	Std Deviation	Variance
Continuous t0	10	82	101	92,90	6,641	44,100
Continuous t30min	10	106	156	134,50	15,479	239,611
Continuous t60min	10	92	169	135,00	28,261	798,667
Continuous t120min	10	100	142	120,50	15,028	225,833
Continuous t180min	10	83	125	109,00	12,463	155,333
Continuous t360min	10	83	93	88,10	3,542	12,544
Continuous 24h	10	85	102	93,50	5,523	30,500
Continuous 48h	10	85	101	92,50	4,790	22,944

Descriptive statistics of blood glucose values (mg/dl) from t = 0 to t = 48h after continuous glucose infusion.

**Table 3:** Descriptive statistics of blood glucose level after bolus infusion.

	N	Min	Max	Mean	Std. Deviation
Bolus t0	10	85	101	93,00	4,761
Bolus t5min	9	153	236	195,78	32,357
Bolus t10min	9	212	308	245,56	31,421
Bolus t15min	9	200	275	225,67	22,506
Bolus t30min	10	117	193	152,50	25,251
Bolus t60min	10	67	137	96,30	18,945
Bolus t65min	8	126	247	171,63	37,743
Bolus t70min	8	161	288	223,25	38,729
Bolus t75min	8	169	296	215,63	41,740
Bolus t120min	10	55	131	82,80	20,395
Bolus t125min	8	95	248	161,63	52,772
Bolus t130min	7	155	322	211,57	67,206
Bolus t135min	8	147	265	198,00	41,210
Bolus t180min	10	59	104	75,50	12,186
Bolus t360min	10	78	92	84,80	4,638
Bolus t24h	9	83	101	90,89	6,936
Bolus t48h	10	77	107	92,60	9,300

Descriptive statistics of blood glucose values (mg/dl) from t = 0 to t = 48h after bolus glucose infusion. Of note, additionally timepoints (t = 5min, t = 10min, t = 15min, t = 65min, t = 70min, t = 75min, t = 125min, t = 130min, t = 135min) are depicted.

Glucose variability was measured by using the coefficient of variation. We calculated the coefficient of variation for every test person on both study days (Table 4).

**Table 4:** Coefficient of variation (in %) for each test person on both study days.

Patient Code	CV Continuous (in %)	CV Bolus (in %)
A	9,65	44,66
B	11,85	21,35
C	16,21	44,26
D	26,48	37,60
E	18,42	40,29
G	27,13	45,09
H	10,81	41,20
I	27,64	41,76
K	12,24	40,91
M	25,49	42,63

Coefficient of variation (in %) for every test person on continuous and bolus study days.

**Fig 5:**
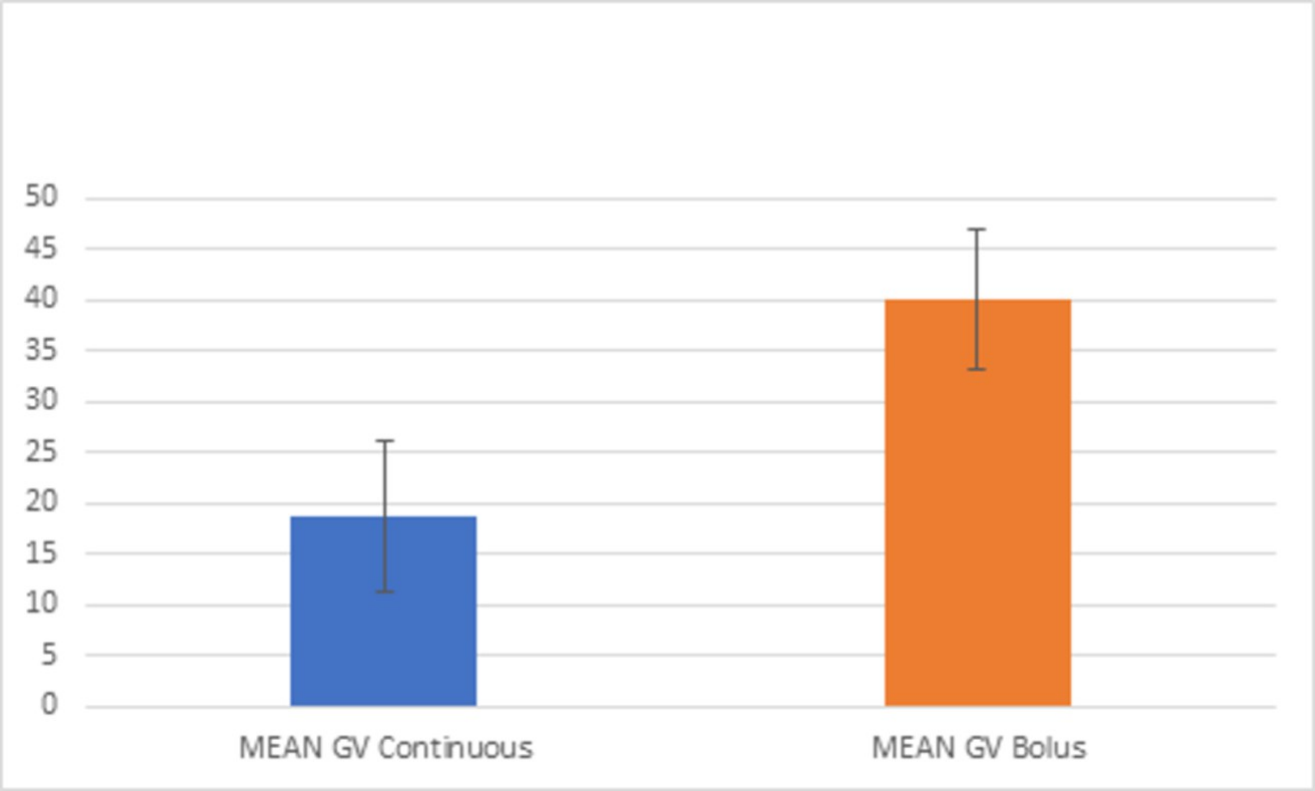
Mean coefficient of variation (in %) for both study days. Mean Coefficient of Variation (in %) for both study days with SD is depicted.

For better representation of glucose variability in between both study days we calculated the mean coefficient of variation for days of continuous glucose infusion and of bolus glucose infusion (Fig 5). The mean CV on days of continuous glucose infusion was 18,59% with a SD of 7,43. Mean CV on days of bolus infusion was 39,98% with a SD of 6,93.

Of the 92 biomarkers analysed in the Cardiovascular II Panel of OLINK, 85 could be utilised for statistical analysis. For 7 biomarkers (STK 4, IL-17D, FGF-23, ITGB1BP2, BNP, CA5A, PARP-1) data was unusable due to missing values and these proteins were excluded from the statistical analysis. Of the remaining 85 biomarkers, 7 biomarkers showed significant differences at t = 360min between the two study days (rapid vs continuous protocol) (Table 5).

**Table 5:** List of cardiovascular biomarkers including p values.

Biomarker		Statistical difference between bolus glucose vs continuous glucose infusion (RM-ANOVA)	Timepoints with significant difference (as calculated by paired t-test or Wilcoxon test)	95% Confidence Interval (CI)	
				Lower Bound	Upper Bound
1. BMP6	Bone morphogenetic protein 6	p = 0,104	360 min, paired t-test, p = 0,033	-0,324	-0,178
2. ANGPT1	Angiopoietin-1	p = 0,184			
3. ADM	ADM	p = 0,126			
4. CD40	CD40 ligand	p = 0,167			
5. SLAMF7	SLAM family member 7	p = 0,096	360 min, Wilcoxon test, p = 0,039	0,007	0,359
6. PGF	Placenta growth factor	p = 0,050			
7. ADAMTS13	A disintegrin and metalloproteinase with thrombospondin motifs, member 13	p = 0,204	360 min, Wilcoxon test, p = 0,020	0,022	0,128
8. BOC	Brother of CDO	p = 0,386			
9. IL4RA	Interleukin-4 receptor subunit alpha	p = 0,147	360min, paired t-test, p = 0,033	-0,306	-0,171
10. SRC	Proto-oncogene tyrosine-protein kinase Src	p = 0,351			
11. IL1RA	Interleukin-1 receptor antagonist protein	p = 0,085	360min, paired t-test, p = 0,044	-0,769	-0,123
12. IL6	Interleukin-6	p = 0,953			
13. TNFRSF10A	Tumor necrosis factor receptor superfamily member 10A	p = 0,186			
14. IDUA	Alpha-L-iduronidase	p = 0,301			
15. TNFRSF11A	Tumor necrosis factor receptor superfamily member 11A	p = 0,170			
16. PAR1	Proteinase-activated receptor1	p = 0,650			
17. TRAILR2	TNF-related apoptosis-inducing ligand receptor 2	p = 0,125			
18. PRSS27	Serine protease 27	p = 0,612			
19. TIE2	Angiopoietin-1 receptor	p = 0,481			
20. TF	Tissue factor	p = 0,355			
21. IL2RL2	Interleukin-1 receptor-like 2	p = 0,556			
22. PDGFsubunit	Platelet-derived growth factor subunit B	p = 0,546			
23. IL27	Interleukin-27	p = 0,157			
24. CXCL1	C-X-C motif chemokine 1	p = 0,587			
25. LOX1	Lectin-like oxidized LDL receptor 1	p = 0,233	360min, paired t-test, p = 0,015	-0,885	-0,125
26. GAL9	Galectin-9	p = 0,307			
27. GIF	Gastric intrinsic factor	p = 0,876			
28. SCF	Stem cell factor	p = 0,700			
29. IL18	Interleukin-18	p = 0,312			
30. FGF21	Fibroblast growth factor 21	p = 0,913			
31. PlgR	Polymeric immunoglobulin receptor	p = 0,793			
32. RAGE	Receptor for advanced glycosylation end products	p = 0,005			
33. SOD2	Superoxide dismutase [Mn], mitochondrial	p = 0,587			
34. CTRC	Chymotrypsin C	p = 0,544			
35. SPON2	Spondin-2	p = 0,790			
36. GH	Growth hormone	p = 0,677			
37. FS	Follistatin	p = 0,046			
38. GLO1	Lactoylglutathione lyase	p = 0,338			
39. CD84	SLAM family member 5	p = 0,217			
40. PAPPA	Pappalysin-1	p = 0,473			
41. SERPINA12	Serpin A12	p = 0,315			
42. REN	Renin	p = 0,452			
43. DCR1	2,4-dienoyl-CoA reductase, mitochondrial	p = 0,177			
44. MERTK	Tyrosine-protein kinase Mer	p = 0,398			
45. KIM1	Kidney injury molecule 1	p = 0,678			
46. THBS2	Thrombospondin-2	p = 0,229			
47. TM	Thrombomodulin	p = 0,578			
48. VSIG2	V-set and immunoglobulin domain containing protein 2	p = 0,686			
49. AMBP	Protein AMBP	p = 0,709			
50. PRELP	Prolargin	p = 0,937			
51. HO-1	Heme oxygenase 1	p = 0,513			
52. XCL1	Lymphotactin	p = 0,039			
53. IL-16	Pro-interleukin-16	p = 0,576			
54. SORT1	Sortilin	p = 0,524			
55. CEACAM8	Carcinoembryonic antigen related cell adhesion molecule 8	p = 0,359			
56. PTX3	Pentraxin-related protein PTX3	p = 0,297	360min, paired t-test, p = 0,033	-0,818	-0,436
57. PSGL1	P-selectin glycoprotein ligand 1	p = 0,896			
58. CCL17	C-C motif chemokine 17	P = 0,241			
59. CCL3	C-C motif chemokine 3	p = 0,239			
60. MMP7	Matrix metalloproteinase-7	p = 0,646			
61. IgGFreceptorIIb	Low affinity immunoglobulin gamma Fc region receptor II-b	p = 0,076			
62. DCN	Decorin	p = 0,176			
63. DKK-1	Dickkopf-related protein 1	p = 0,363			
64. LPL	Lipoprotein lipase	p = 0,020			
65. PRSS8	Prostasin	p = 0,273			
66. AGRP	Agouti-related protein	p = 0,458			
67. HB-EGF	Proheparin-binding EGF-like growth factor	p = 0,631			
68. GDF-2	Growth/differentiation factor 2	p = 0,224			
69. FABP2	Fatty acid-binding protein, intestinal	p = 0,244			
70. THPO	Thrombopoietin	p = 0,446			
71. MARCO	Macrophage receptor MARCO	p = 0,652			
72. GT	Gastrotropin	p = 0,848			
73. MMP12	Matrix metalloproteinase-12	p = 0,535			
74. ACE2	Angiotensin-converting enzyme 2	p = 0,148			
75. PD-L2	Programmed cell death 1 ligand 2	p = 0,174			
76. CTSL1	Cathepsin L1	p = 0,346			
77. hOSCAR	Osteoclast-associated immunoglobulinlike receptor	p = 0,587			
78. TNFRSF13B	Tumor necrosis factor receptor superfamily member 13B	p = 0,639			
79. TGM2	Protein-glutamine gammaglutamyltransferase 2	p = 0,236			
80. LEP	Leptin	p = 0,371			
81. HSP27	Heat shock 27 kDa protein	p = 0,701			
82. CD4	T-cell surface glycoprotein CD4	p = 0,519			
83. NEMO	NF-kappa-B essential modulator	p = 0,503			
84. VEGFD	Vascular endothelial growth factor D	p = 0,897			
85. HAOX1	Hydroxyacid oxidase 1	p = 0,755			
**Excluded biomarkers:**		**Excluded due to:**			
86. STK 4	Serine/threonine-protein kinase 4	Missing values			
87. IL-17D	Interleukin-17D	Missing values			
88. FGF-23	Fibroblast growth factor 23	Missing values			
89. ITGB1BP2	Melusin	Missing values			
90. BNP	Natriuretic peptides B	Missing values			
91. CA5A	Carbonic anhydrase 5A, mitochondrial	Missing values			
92. PARP-1	Poly [ADP-ribose] polymerase 1	Missing values			

List of analysed biomarkers (Cardiovascular II Panel, OLINK) including p values.

**Fig 6:**
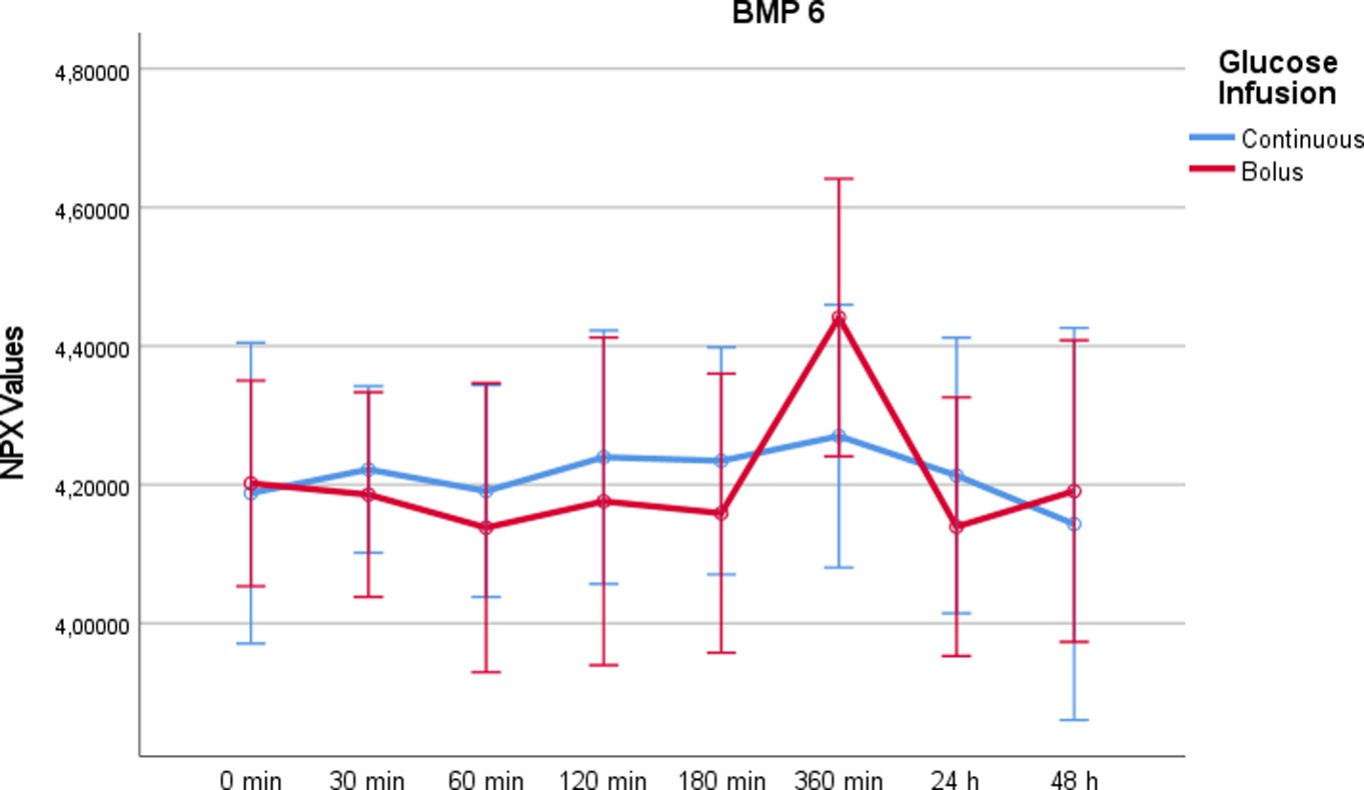
BMP6 values on a NPX scale after administration of rapid glucose (bolus) vs. continuous glucose. Whereas RM-ANOVA did not reveal significant differences between continuous and bolus administration of glucose (p = 0,104), there is a marked increase at 360 minutes after bolus administration, which was statistically significant using paired t-test (p = 0,033). Of note, the NPX format is an arbitrary log2 unit. Therefore, the depicted values do not reflect actual concentrations.
- Interleukin-4 receptor subunit alpha (IL-4RA)
  After both continuous and rapid administration of glucose, IL-4RA showed an increase after 24h and 48h compared to the beginning of the measurements (Fig 7). Overall there was no significant difference between the two study days (p = 0,147). By paired t-test at t = 360min, there was a significant increase with rapid glucose infusion compared to continuous administration (p = 0.033).

The focus of our analysis was therefore on the following biomarkers:

- Bone morphogenetic protein 6 (BMP-6)
  Comparing the two study days, BMP-6 showed no relevant increase during and after the administration of glucose (p = 0,104, as calculated by RM-ANOVA), whereas at time point t = 360min a relevant increase of the protein was observed after rapid glucose administration (Fig 6). The increase was not detectable after 24h or 48h. Using a paired t-test for the time point t = 360min, a significant difference was found (p = 0.033).

**Fig 7:**
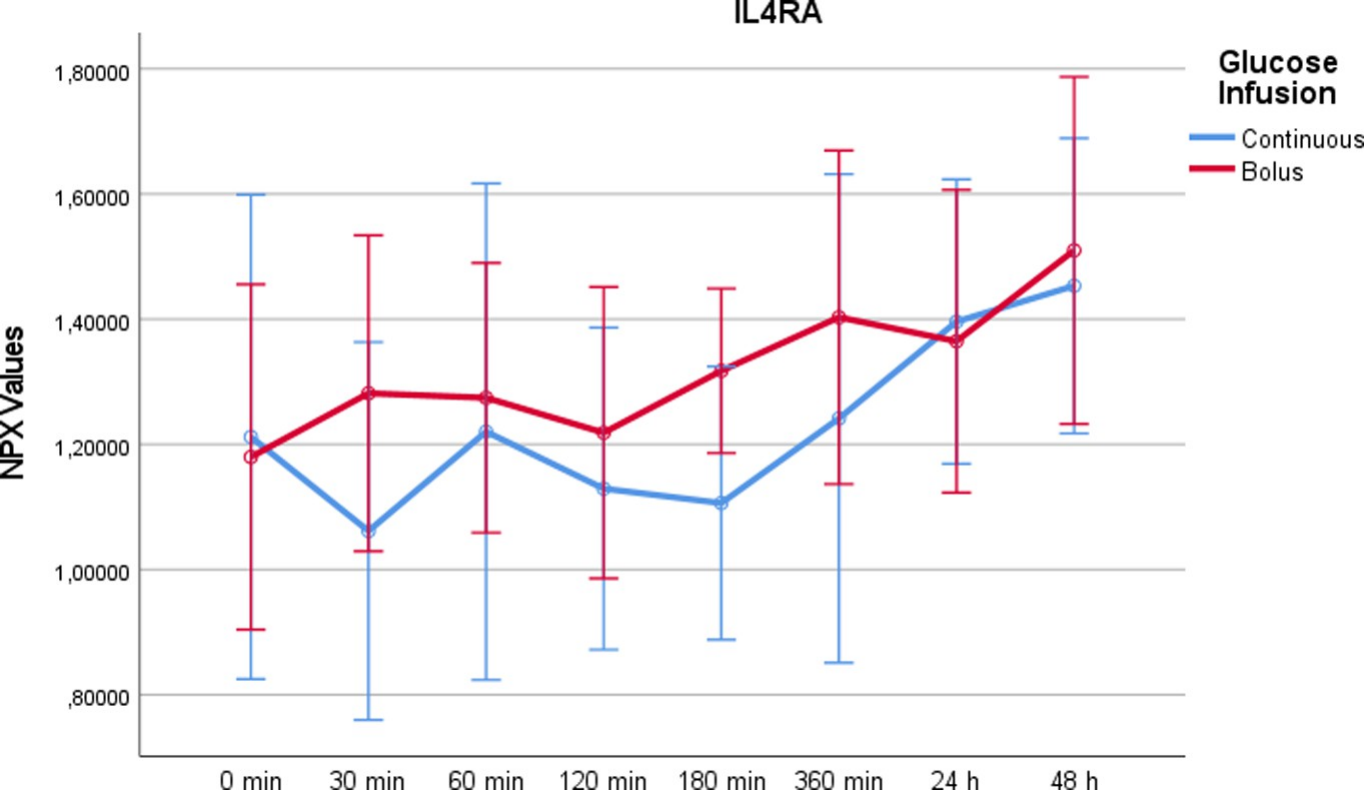
IL4RA values on a NPX scale after administration of rapid glucose (bolus) vs. continuous glucose. Whereas RM-ANOVA did not reveal significant differences between continuous and bolus administration of glucose (p = 0,147), there is a significant difference at 360 minutes after bolus administration regarding paired t-test (p = 0,033). Of note, the NPX format is an arbitrary log2 unit. Therefore, the depicted values do not reflect actual concentrations.
- Pentraxin-related protein PTX3 (PTX3)
  While there was no significant change between the two study days for PTX3 (p = 0,297), a significant increase was seen at time point t = 360min with rapid glucose infusion in comparison to continuous infusion (p = 0.033) (Fig 8).

**Fig 8:**
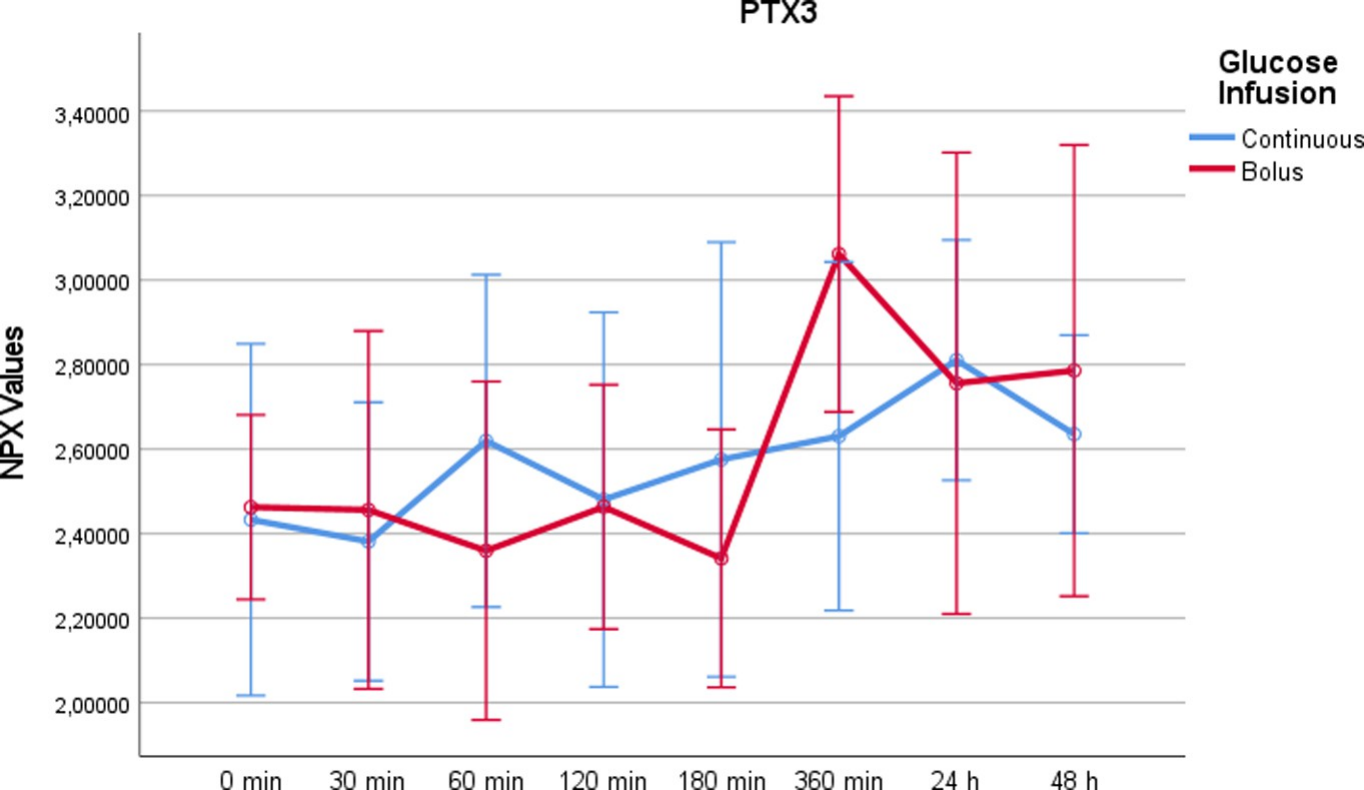
PTX3 values on a NPX scale after administration of rapid glucose (bolus) vs. continuous glucose. Whereas RM-ANOVA did not reveal significant differences between continuous and bolus administration of glucose (p = 0,297), there is a marked increase at 360 minutes after bolus administration, which was statistically significant using paired t-test (p = 0,033). Of note, the NPX format is an arbitrary log2 unit. Therefore, the depicted values do not reflect actual concentrations.
- Lectin-like oxidised LDL receptor 1 (LOX-1)
  After rapid glucose infusion, there was a significant increase at t = 360min compared to continuous administration (p = 0.015) (Fig 9). There were no significant differences between the two study days (p = 0,233).

**Fig 9:**
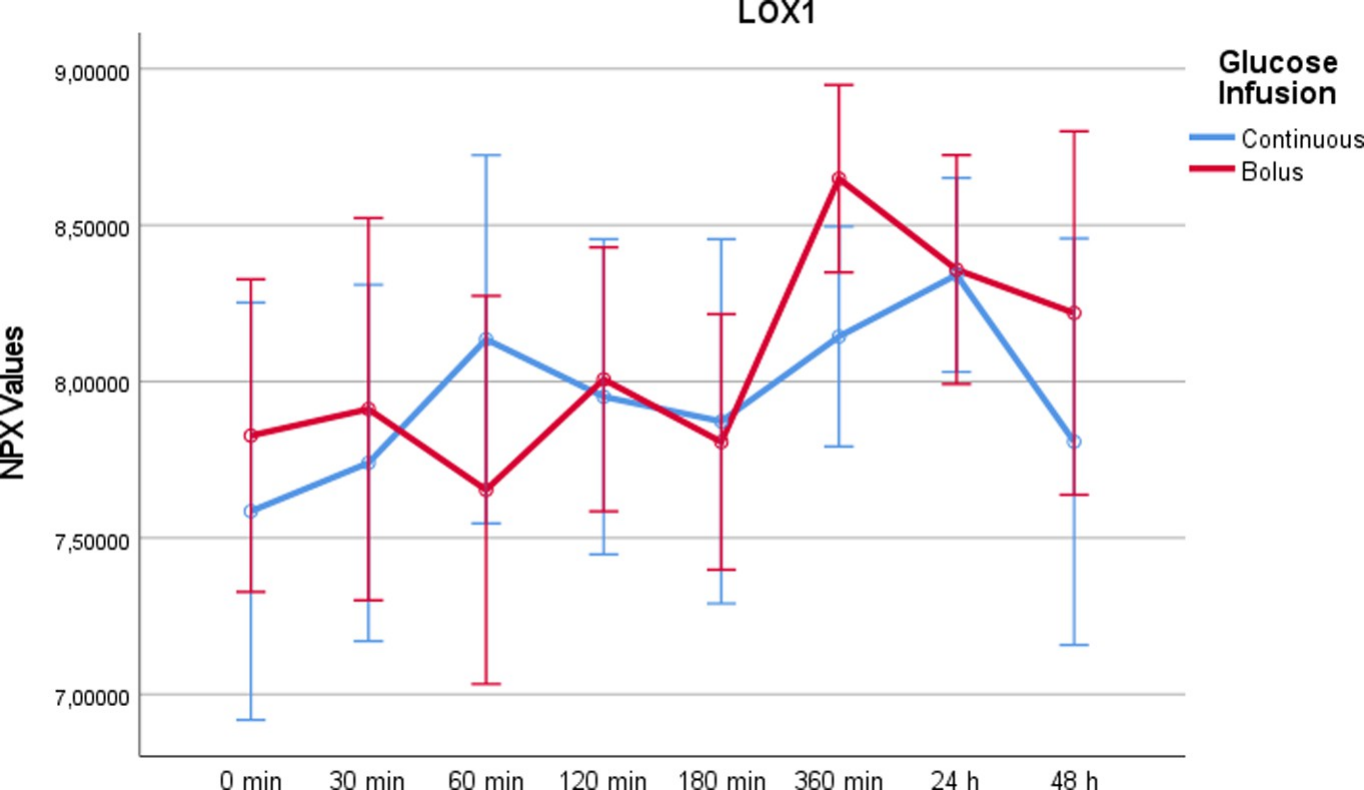
LOX1 values on a NPX scale after administration of rapid glucose (bolus) vs. continuous glucose. Whereas RM-ANOVA did not reveal significant differences between continuous and bolus administration of glucose (p = 0,233), there is a marked increase at 360 minutes after bolus administration, which was statistically significant using paired t-test (p = 0,015). Of note, the NPX format is an arbitrary log2 unit. Therefore, the depicted values do not reflect actual concentrations.
- Interleukin-1 receptor antagonist protein (IL-1RA)
  IL-1RA showed no significant increase or decrease after continuous glucose administration, whereas there was a significant upward deflection after rapid glucose infusion at t = 360min (p = 0.044) (Fig 10). There were no significant differences comparing the two study days (p = 0,085).

**Fig 10:**
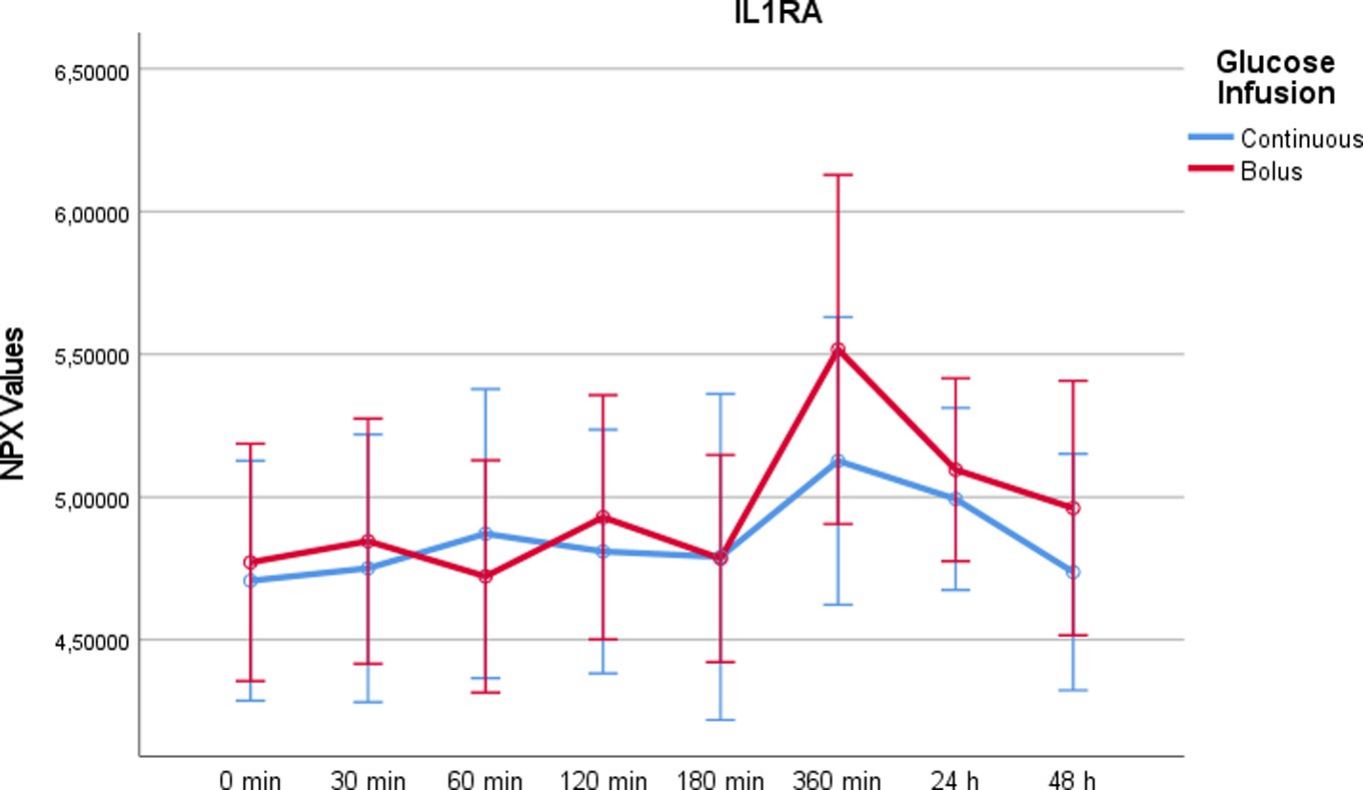
IL1RA values on a NPX scale after administration of rapid glucose (bolus) vs. continuous glucose. Whereas RM-ANOVA did not reveal significant differences between continuous and bolus administration of glucose (p = 0,085), there is a marked increase at 360 minutes after bolus administration, which was statistically significant using paired t-test (p = 0,044). Of note, the NPX format is an arbitrary log2 unit. Therefore, the depicted values do not reflect actual concentrations.
- A Disintegrin and Metalloproteinase with a thrombospondin type I motif, member 13 (ADAMTS13)
  At time point t = 360min, there was a significant increase in ADAMTS13 following rapid glucose infusion compared to continuous glucose infusion using Wilcoxon test (p = 0,020) (Fig 11). There were no significant differences at the other timepoints (p = 0,204).

**Fig 11:**
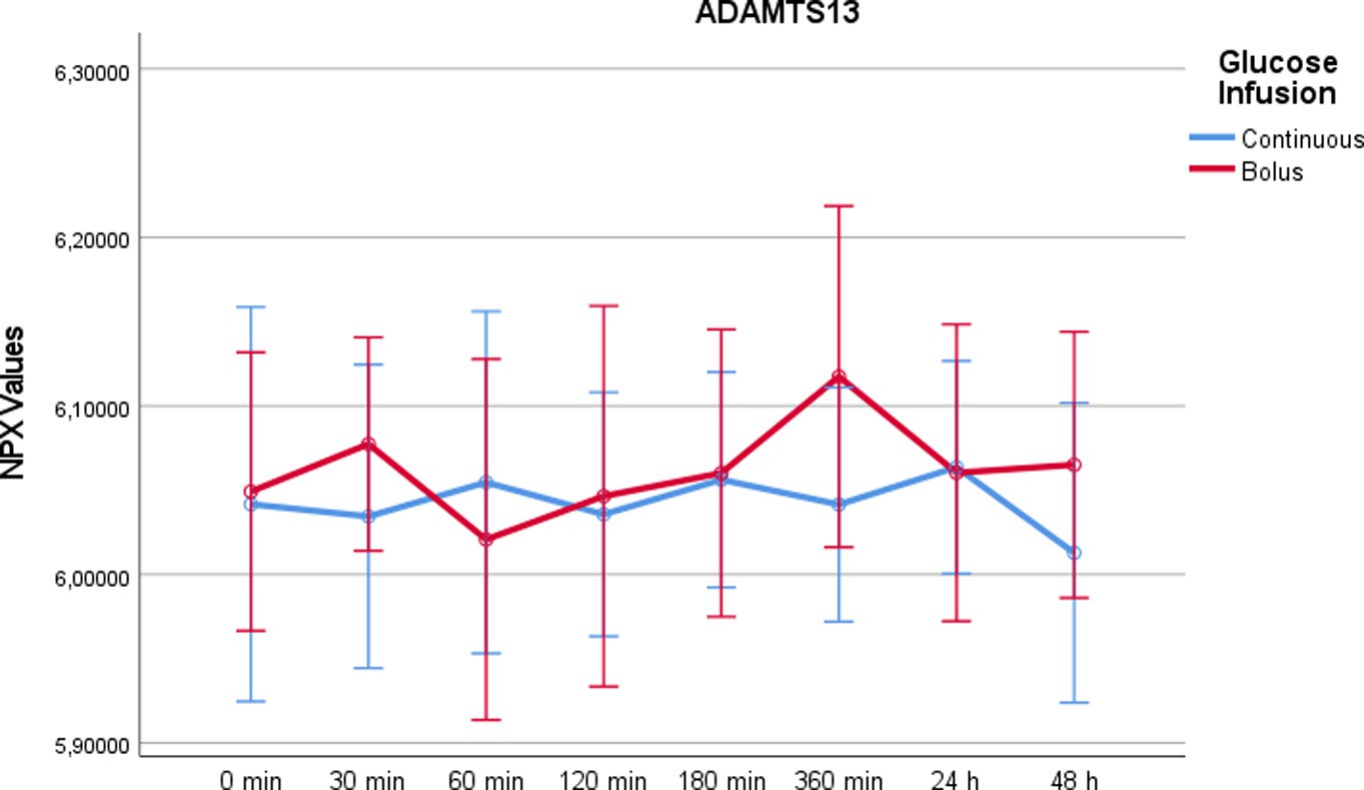
ADAMTS13 values on a NPX scale after administration of rapid glucose (bolus) vs. continuous glucose. Whereas RM-ANOVA did not reveal significant differences between continuous and bolus administration of glucose (p = 0,204), there is a marked increase at 360 minutes after bolus administration, which was statistically significant using Wilcoxon test (p = 0,020). Of note, the NPX format is an arbitrary log2 unit. Therefore, the depicted values do not reflect actual concentrations.
- SLAM family member 7 (SLAMF7)
  From time point t = 360min onwards, SLAMF7 showed higher values in the rapid glucose intake group. The difference at t = 360min was significant between the two study days using Wilcoxon test (p = 0.039) (Fig 12). Overall there was no significant difference between the two study days (p = 0,096).

**Fig 12:**
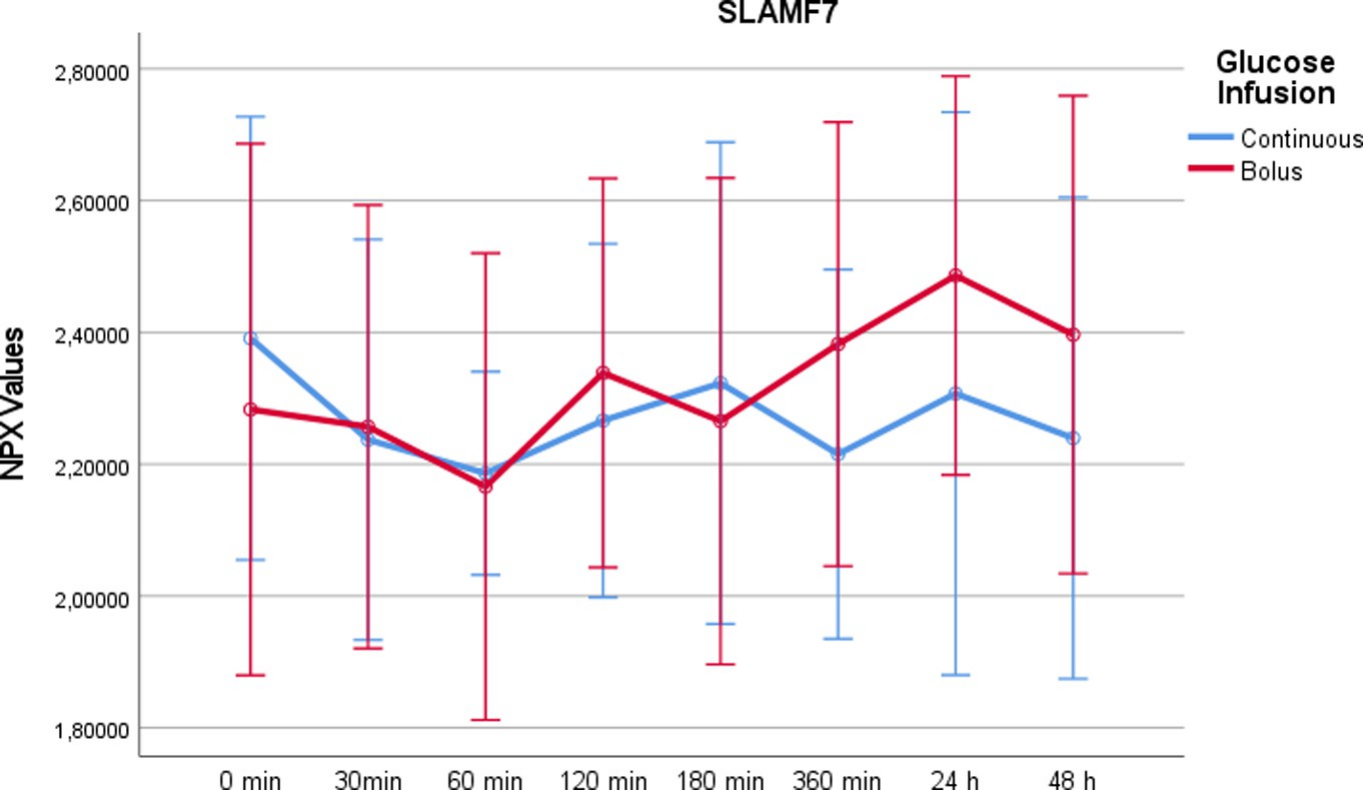
SLAMF7 values on a NPX scale after administration of rapid glucose (bolus) vs. continuous glucose. Whereas RM-ANOVA did not reveal significant differences between continuous and bolus administration of glucose (p = 0,096), there is an increase at 360 minutes after bolus administration, which was statistically significant using Wilcoxon test (p = 0,039). Of note, the NPX format is an arbitrary log2 unit. Therefore, the depicted values do not reflect actual concentrations.

## Discussion

Diabetes mellitus increases cardiovascular mortality 2 to 3-fold compared to a healthy control population via microvascular and macrovascular complications [1, 2].

The aim of our study was to evaluate the effects of rapid glucose excursions by measuring the difference between bolus glucose infusion compared to slow continuous glucose infusion in a broad panel of 92 biomarkers involved in cardiovascular processes.

In a similarly designed study, administration of LPS, which induces sepsis, showed significantly stronger effects on cardiovascular biomarkers in comparison to the experimental glucose excursions in this study [26]. It was expected that glucose administration (both bolus and continuous) would have a much smaller effect on cardiovascular biomarkers than artificial inflammation exerted by LPS administration. In this respect, the focus of our study was to detect those biomarkers that already react measurably to short-term changes in blood glucose levels.

Our measurements showed significant changes in seven biomarkers in the comparison between rapid and slow glucose intake, which will be examined in more detail below.

**Bone morphogenetic protein 6 (BMP6).** The effects of bone morphogenetic proteins (BMPs) range from bone and cartilage formation to influences on cellular differentiation and organ development. In macrophage cell lines, it was shown in mice that cells treated with BMP6 had a similar morphology to cells activated by LPS (lipopolysaccharide). This may indicate the role of BMP6 as a potential regulator of macrophages [27].

In another study, mice were treated with BMP-6 and it was proven that during the 6-day treatment plasma glucose levels were lowered and glucose excursions during an OGTT were minimised [28].

In patients with chronic heart failure, levels of BMP6 were elevated compared to controls, with higher levels in patients of more advanced disease [29].

Furthermore, in another study BMP6 was shown to be a significant biomarker for the development of coronary heart disease—patients with low BMP6 levels showed an increased risk of developing CHD [30].

**Pentraxin-related protein PTX3 (PTX3).** PTX3, together with other acute phase proteins (such as CRP) is a member of the pentraxins superfamily. While under normal circumstances there are low concentrations in the blood of healthy subjects, PTX3 levels increase rapidly during inflammation [31]. Generally speaking, in studies high levels of PTX3 have been associated with an unfavourable outcome (and increased mortality) and PTX3 was elevated in systemic inflammatory response syndrome and septic shock [32, 33]. In the first days during bacteraemia in patients, PTX3 was even superior to CRP as a prognostic marker [31]. It should be mentioned that PTX3 initiates both protective and harmful processes and accordingly gives contradictory results in studies [34, 35].

PTX3 is likely to be an independent risk factor for the development of vascular events and atherosclerosis [36]. After myocardial infarction, PTX3 plasma levels peaked at 7.5 h, providing evidence that PTX3 is a strong prognostic marker for cardiovascular mortality [36].

In studies with patients suffering from gestational diabetes, a connection is suspected between PTX3 and the pathophysiology of GDM [37]. Furthermore, a significant correlation was found between the maternal sera of PTX3 levels and high blood glucose levels [38].

**Lectin-like oxidised LDL receptor 1 (LOX-1).** LOX-1 represents one of several scavenger receptors that play an important role in the formation of atherosclerosis via oxidised LDL (ox-LDL). LOX-1 itself is a transmembrane glycoprotein that binds and takes up ox-LDL [39].

Studies have shown SLox-1 (soluble LOX-1), a measurable fragment of the LOX-1 molecule, to increase as a diagnostic marker in patients with coronary artery disease, diabetes, hypertension and metabolic syndrome [39]. Thus, an increased expression of LOX-1 was shown in inflammatory processes, which could not be detected under physiological conditions [40, 41].

Further studies indicated that LOX-1 was elevated in the setting of acute coronary syndromes and showed faster increases than troponin T [42, 43].

LOX-1 was found to be significantly higher in biopsies of epicardial adipose tissue in patients with type 2 diabetes and ischemic heart disease compared to control groups without type 2diabetes mellitus. No significant association was found between the duration of diabetes and increased LOX-1 [44].

It has already been shown in vitro that high glucose concentrations can induce the expression of endothelial LOX-1 [45].

**Interleukin-1 receptor antagonist protein (IL-1Ra).** There is a close relationship between IL-1α, IL-1β - which increases nitrooxidative stress—and IL-1Ra in the activation and suppression of inflammation in the body [46, 47].

According to studies, the administration of an IL-1 receptor antagonist (anakinra) reduced nitrooxidative stress and improved cardiac left ventricular function compared to control groups [47].

Higher plasma IL-1RA concentrations are associated with an increased risk of developing type 2 diabetes mellitus, whereas in manifest diabetes IL-1RA concentrations are lower compared to controls. It is possible that the higher concentrations represent attempts by the organism to prevent the harmful processes of IL-1 β and to maintain both insulin production and insulin sensitivity [48].

In studies, the administration of recombinant IL-1RA (anakinra) over several weeks was not only able to stop inflammatory processes but also to reduce HbA1c values by 0.5% compared to the placebo group [49, 50].

**ADAMTS13.** ADAMTS13 is a metalloproteinase that is primarily synthesized in the liver. One of its major functions is cleaving Von Willebrand Factor protein (vWF) [51]. Due to its antithrombotic properties, low ADAMTS13 activity seems to be a risk factor for the development of ischemic stroke and myocardial infarction [52, 53].

Apart from its role as a regulator of thrombosis, ADAMTS13 also seems to have an impact on inflammatory processes and angiogenesis [51].

Low ADAMTS13 levels have been associated with increased mortality in patients suffering from sepsis or in septic shock [54].

In the plasma of patients with diabetes some studies have seen significantly lower concentrations of ADAMTS13 activity compared to control groups [55]. In other studies, the ADAMTS13 activity was higher in patients with type 2 diabetes [56]. In the same study ADAMTS13 activity was furthermore associated with a higher risk of incident diabetes and suggested that ADAMTS13 has a role in the occurrence of type 2 diabetes at earlier stages–even before a rise in glucose [56].

**Signalling lymphocytic activation molecule-F7 (SLAMF7).** Studies have shown that SLAMF7 is overexpressed on multiple myeloma cell surfaces in studies and is therefore considered a target of multiple myeloma therapy [57].

SLAMF7 as a biomarker could also provide information on the progression of multiple myeloma [58].

**Interleukin-4 receptor subunit alpha (IL-4Rα).** As Interleukin-4 or Interleukin-13 bind to the Interleukin-4 receptor on macrophages, this leads to macrophage activation. IL-4Rα is one component of the interleukin-4 receptor [59, 60].

Interleukin 4 has been linked in studies to allergic reactions as well as inflammatory and malignant processes [59].

### Limitations

The relatively small number of participants is a possible limitation of our study. However, due to the cross-over design with all subjects receiving both continuous as well as bolus glucose infusions on two different study days, sufficient power to identify biomarkers influenced by glucose excursions can be assumed. Importantly, this study integrated male, healthy subjects only; female subjects were excluded due to potential hormonal effects on cardiovascular biomarkers during menstruation.

Another limitation of this study is the design regarding glucose intake—we chose to administer 20 grams of glucose i.v. three times compared to 1x60 grams of glucose i.v.. It seems possible that higher doses and thus greater rapid glucose excursions would show stronger effects on the biomarkers of interest.

### Impact

The effects of glucose variability on cardiovascular processes are currently still the subject of controversy. Although there are already some studies providing evidence that glucose variability (independent of HbA1c) is a relevant risk factor for the development of cardiovascular complications, there is still a lack of clear definitions of how glucose variability should be measured and described.

In this study, we have focused on glucose variability intraday, i.e. within a short period of time. It was surprising for us that even such short-acting changes in blood glucose levels, which certainly could not be reflected in HbA1c measurements, had an effect on some of the biomarkers already described in detail here.

Thus two major conclusions can be drawn from our work.

First, the measurement of HbA1c reflects only part of the risk on cardiovascular processes.

Second, even small and short-term excursions in blood glucose levels can induce an effect on cardiovascular biomarkers in healthy subjects without diabetes mellitus.

The PCR measurements of the broad panels available now make it possible to measure the effects of glucose variability on many biomarkers that are integrated in a variety of processes.

## Conclusion

In this prospective study, we have been able to show for the first time that rapid glucose administration of 3x20g glucose i.v. compared to the continuous administration of 1x60g i.v. in healthy male volunteers has effects on cardiovascular biomarkers and has shown significant changes in BMP6, SLAMF7, ADAMTS13, IL1RA, PTX3, IL-4RA and LOX-1.

## Supporting information

S1 Checklist Consort 2010 checklist of information. (DOC)

S1 File Study protocol. (DOCX)

S1 Data Dataset including cardiovascular biomarkers (NPX values) and glucose values. (XLSX)

## Abbreviations

BMP6: Bone morphogenetic protein 6
SLAMF7: SLAM family member 7
LOX-1: Lectin-like oxidised LDL receptor 1
ADAMTS13: A Disintegrin and Metalloproteinase with a thrombospondin type I motif, member 13
IL-1RA: Interleukin-1 receptor antagonist protein
IL-4RA: Interleukin-4 receptor subunit alpha
PTX3: Pentraxin-related protein PTX3
GV: Glycaemic variability
