# Supplementary material for: Effect of repeated bolus and continuous glucose infusion on a panel of circulating biomarkers in healthy volunteers
Source: PLoS One. 2022 Dec 27;17(12):e0279308. doi: 10.1371/journal.pone.0279308 (PMC9794098; doi:10.1371/journal.pone.0279308)
Supplement: S1 File — (DOCX) [file pone.0279308.s002.docx]

**Effect of rapid glucose excursion versus continuous glucose infusion on cardiovascular and metabolic parameters in healthy volunteers**

**Effekt der Glucose-Variabilität auf endokrine und kardiovaskuläre Parameter**

M. Resl, M. Heinzl, C. Klammer, M. Clodi

KH der Barmherzigen Brüder Linz

Saturday, 09 February 2019

**Introduction**

At the end of the 20th century, it became known that people with diabetes have a five times higher risk of dying of cardiovascular death than non-diabetics. This has been demonstrated in epidemiological studies as well as in the United Kingdom Prospective Diabetes Study (UKPDS). ^1,2^

However, studies that compared a good blood glucose control with a less intensive one have shown no cardiovascular benefit (UKPDS, ACCORD, ADVANCE, DCCT). These studies covered a study period of five years on average. After the end of these studies, the patients were nevertheless followed for many years. In these years, all patients had the same glucose control. Fortunately, it was then shown that the patients who were in the intensive group during the study period had a positive cardiovascular effect at follow up. This effect is now referred to as “glucose memory” (UKPDS follow up, ACCORDION, ADVANCE-ON, EDIC).

In addition to these indisputable proofs that chronic hyperglycaemia plays a role in the pathogenesis of cardiovascular diseases and glucose lowering therapy might have a benefit, glycaemic variability has recently also been regarded as another risk factor for cardiovascular and microvascular complications^3-7^. Furthermore it was shown that glucose variability cannot be adequately represented by HbA1c, the gold standard for assessment of glucose control.^8^ The term glycaemic or glucose variability (GV) refers to fluctuations in the blood sugar level. The cause is assumed to be a reduced or lacking self-regulation or incorrect drug self-control. Intermittent blood sugar excursions with pronounced fluctuations between high and low values instead of constant, even increased blood sugar exposure, have been shown to be more harmful in several studies.^9-12^ For example, a recent study found an association between glucose variability and autonomous cardiovascular neuropathy (CAN) independent of mean glucose, even after adaptation to other clinical risk factors of CAN such as HbA1c.^7^ A further study showed that glucose variability early after the onset of ACS is a predictor of rapid progression of non-culprit lesions in coronary arteries.^13^

Glycaemic variability may therefore be a factor in the development of diabetic complications, but there is a lack of conclusive evidence. The cause of these negative influences on the cardiovascular system due to high glucose variability has not been sufficiently investigated and is therefore not fully understood so far. Unfortunately have all available glycaemic biomarkers, including A1C, limitations and should therefore not be used for interpretation of glycaemic variability. The recent clinical application of continuous glucose measurement (CGM) has enabled to begin a detailed assessment of the glycaemic variability.^14^ By using CGM we can further understand the damage caused by glucose variability.

So showed one recent study that glycaemic variability correlated to oxidized low density lipoprotein, E-selectin, intercellular adhesion molecule 1, C-reactive protein and soluble receptor for advanced glycosylation end product in healthy and diabetic adolescents^15^ and another group showed a reduction in CRP via reduction of glycaemic variability.^16^ In another study in mice glycaemic variability significantly increased the expression levels of proinflammatory cytokines TNF-alpha, IL-6 and of NF-kappaB.^17^

To date, however, there have been no studies with acutely altered glucose concentrations in healthy volunteers that have investigated the effects on the now additionally known cardiovascular and metabolic biomarkers. New techniques in analysis provide scientists with the opportunity to use explorative panels to identify a broader range of new biomarkers and relevant protein signatures that may reflect important biological processes and may have far-reaching clinical relevance. The cardiometabolic panel to be used here provides simultaneous analysis of 92 protein biomarkers. The selection of protein biomarker assays is designed to focus on proteins relevant for cardiometabolic processes. The assays in this panel include proteins involved in important biological processes such as cellular metabolic process, cell adhesion, immune response and complement activation.^18^

The aim of this study is to determine the effect of rapid glucose excursion versus continuous glucose infusion on cardiovascular and metabolic parameters in healthy volunteers.

**Subjects**

Before the study all ten male subjects will be screened by history, physical examination, and electrocardiogram.

The study will be approved by the local joint research ethics committee of the “Krankenanstalten Krankenhaus der Barmherzigen Schwestern Linz und Konventhospital der Barmherzige Brüder Linz” and informed consent will be obtained in writing from each subject before enrolment in the study.

**Inclusion/exclusion criteria:**

Eligible patients must meet the following inclusion criteria:

- Men aged 18 years or older with no disease history
- written informed consent

Eligible patients must meet none of the following exclusion criteria:

- receiving any medication
- probands who suffer from infectious disease

**Study protocol**

Each subject will be studied on two occasions, 7–21 days apart. In random order, the subjects will receive 3 times 20 grams glucose intravenously over 5 minutes at intervals of one hour or continuously over 3 hours 60 grams glucose.

A Dexcom G4 continuous glucose monitoring systems will be set up one day before the scheduled study days and subjects will be asked to carry the system for 48 hours after application.

In between studies subjects consumed a weight-maintaining diet providing at least 200 g carbohydrate each day. All subjects will be studied at 0830 h after an overnight fast and refrained from smoking or caffeine-containing beverages for 24 h before the study. After voiding, subjects will be weighed to the nearest 0.1 kg and will be measured to the nearest 1 cm using a calibrated scale (Seca Ltd.). The subjects, lightly clothed, then rested quietly in a supine position for the remainder of the study. Catheters (Safety iv Catheter with injection port; Braun, Melsungen, Germany) will be inserted into a vein of the right arm (for infusion) and into a vein of the left hand (for sampling). After a 25-min rest period subjects will receive 3 times 20 grams glucose dissolved in 100 ml of a 0.9% NaCl solution intravenously over 5 minutes in intervals of one hour or continuously over 3 hours 60 grams of glucose solved in 300 ml of 0.9 % NaCl solution.

Blood will be taken for repeated measurements of hormone (ACTH, cortisol, GH, IGF-1, Glucagon), cytokine (TNF-alfa, IL-1ß, IL-6) and cardiovascular parameters (OLINK cardiovascular panel) every 30 minutes over the following 6 h outlined below and after 24 hours. At the end of each study, subjects void again, and urine volume will be measured. A 10-mL aliquot of urine will be stored at -4 C for later analysis.

**Timetables**


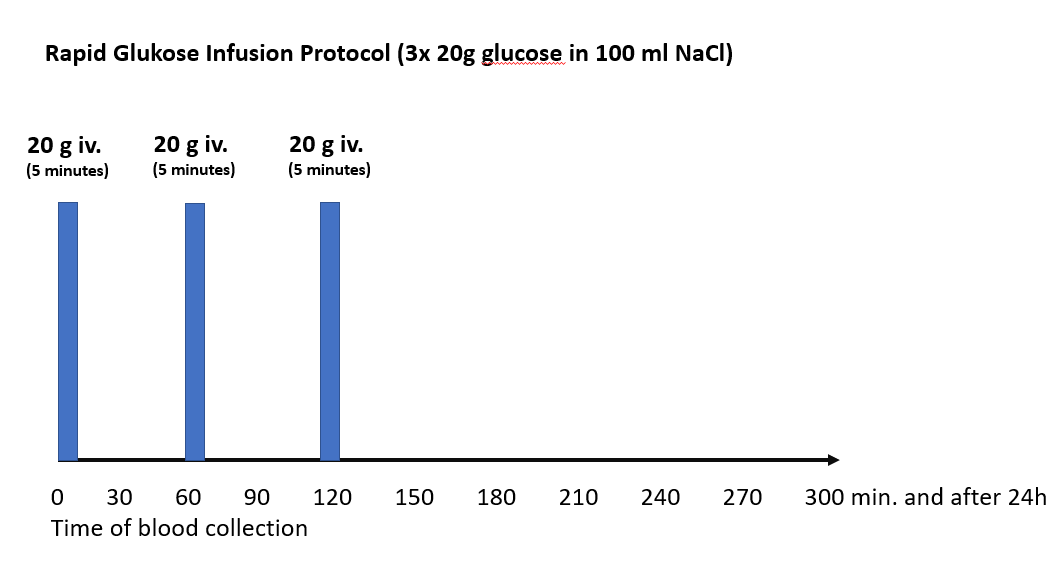


**Sample collection**

Venous blood samples will be taken every 30 minutes and after 24 hours for measurements of hormone, cytokine concentrations and olink panel collection. Samples will be centrifuged, and the plasma will be separated and stored immediately at -20 C

**Biochemical analysis**

Hormones. Commercially available RIA kits will be used for the measurement.

Cytokines. Plasma TNF
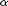
 and IL-6, IL 1ß concentrations will be measured by immunoenzymometric assays.

Cardiometabolic Panel will be measured using OLINK cardiometabolic plates.

**Temperature, pulse, and blood pressure measurement**

Tympanic and axillary membrane temperature will be measured at 15-min intervals using an infrared probe. Pulse rate and mean arterial pressure (MAP) will be measured automatically every 15 min using an electronic electrocardiographic and sphygmomanometric monitor .

**Statistical analysis**

ANOVA for repeated measures will be used to assess time effects and treatment-time interactions. When this reveals a significant treatment-time interaction, *post-hoc* pairwise comparison between rapid glucose infusion and continuous infusion groups will be made using Student’s *t* test with correction for multiple comparisons where appropriate. All calculations will be performed using Statistical Package for the Social Sciences computer software (SPSS).

REFERENCES

1. Stephens JW, Ambler G, Vallance P, Betteridge DJ, Humphries SE, Hurel SJ. Cardiovascular risk and diabetes. Are the methods of risk prediction satisfactory? *Eur J Cardiovasc Prev Rehabil.* 2004;11(6):521-528.

2. Mahmood SS, Levy D, Vasan RS, Wang TJ. The Framingham Heart Study and the epidemiology of cardiovascular disease: a historical perspective. *Lancet.* 2014;383(9921):999-1008.

3. Borg R, Kuenen JC, Carstensen B, et al. HbA(1)(c) and mean blood glucose show stronger associations with cardiovascular disease risk factors than do postprandial glycaemia or glucose variability in persons with diabetes: the A1C-Derived Average Glucose (ADAG) study. *Diabetologia.* 2011;54(1):69-72.

4. Kim MK, Han K, Park YM, et al. Associations of Variability in Blood Pressure, Glucose and Cholesterol Concentrations, and Body Mass Index With Mortality and Cardiovascular Outcomes in the General Population. *Circulation.* 2018;138(23):2627-2637.

5. Liang S, Yin H, Wei C, Xie L, He H, Liu X. Glucose variability for cardiovascular risk factors in type 2 diabetes: a meta-analysis. *J Diabetes Metab Disord.* 2017;16:45.

6. Tang X, Li S, Wang Y, et al. Glycemic variability evaluated by continuous glucose monitoring system is associated with the 10-y cardiovascular risk of diabetic patients with well-controlled HbA1c. *Clin Chim Acta.* 2016;461:146-150.

7. Jun JE, Lee SE, Lee YB, et al. Continuous glucose monitoring defined glucose variability is associated with cardiovascular autonomic neuropathy in type 1 diabetes. *Diabetes Metab Res Rev.* 2019;35(2):e3092.

8. Ceriello A, Ihnat MA. 'Glycaemic variability': a new therapeutic challenge in diabetes and the critical care setting. *Diabet Med.* 2010;27(8):862-867.

9. Jones SC, Saunders HJ, Qi W, Pollock CA. Intermittent high glucose enhances cell growth and collagen synthesis in cultured human tubulointerstitial cells. *Diabetologia.* 1999;42(9):1113-1119.

10. Horvath EM, Benko R, Kiss L, et al. Rapid 'glycaemic swings' induce nitrosative stress, activate poly(ADP-ribose) polymerase and impair endothelial function in a rat model of diabetes mellitus. *Diabetologia.* 2009;52(5):952-961.

11. Piconi L, Quagliaro L, Assaloni R, et al. Constant and intermittent high glucose enhances endothelial cell apoptosis through mitochondrial superoxide overproduction. *Diabetes Metab Res Rev.* 2006;22(3):198-203.

12. Quagliaro L, Piconi L, Assaloni R, Martinelli L, Motz E, Ceriello A. Intermittent high glucose enhances apoptosis related to oxidative stress in human umbilical vein endothelial cells: the role of protein kinase C and NAD(P)H-oxidase activation. *Diabetes.* 2003;52(11):2795-2804.

13. Kataoka S, Gohbara M, Iwahashi N, et al. Glycemic Variability on Continuous Glucose Monitoring System Predicts Rapid Progression of Non-Culprit Lesions in Patients With Acute Coronary Syndrome. *Circ J.* 2015;79(10):2246-2254.

14. Chon S. How can we easily measure glycemic variability in diabetes mellitus? *Diabetes Metab J.* 2015;39(2):114-116.

15. Dasari PS, Gandomani BS, Teague AM, Pitale A, Otto M, Short KR. Glycemic Variability Is Associated with Markers of Vascular Stress in Adolescents. *J Pediatr.* 2016;172:47-55 e42.

16. Schnell O, Amann-Zalan I, Jelsovsky Z, et al. Changes in A1C levels are significantly associated with changes in levels of the cardiovascular risk biomarker hs-CRP: results from the SteP study. *Diabetes Care.* 2013;36(7):2084-2089.

17. Yang J, Zhao Z, Yuan H, et al. The mechanisms of glycemic variability accelerate diabetic central neuropathy and diabetic peripheral neuropathy in diabetic rats. *Biochem Biophys Res Commun.* 2019;510(1):35-41.

18. OLINK. <https://www.olink.com/products/cardiometabolic-panel/>. *Cardiometabolic Panel.* 2019.
